# The Role of Adjunctive Medial Plating in Distal Femur Fractures and Distal Femur Fractures Nonunions

**DOI:** 10.7759/cureus.78538

**Published:** 2025-02-05

**Authors:** Jerrod Steimle, Jaron L Lohmeyer, Benjamin Taylor, Daniel T DeGenova

**Affiliations:** 1 Orthopaedic Surgery, Kettering Health Network, Dayton, USA; 2 Orthopaedic Trauma, OhioHealth Grant Medical Center, Columbus, USA; 3 Orthopaedic Surgery, OhioHealth Grant Medical Center, Columbus, USA

**Keywords:** distal femur fracture, distal femur nonunion, dual plate construct, locked lateral plating, nail plate

## Abstract

Purpose: There exists controversy in the treatment of acute distal femur fractures as well as distal femur fracture nonunions. The objective of this study is to determine the clinical benefit of adjunctive medial plate application in the setting of acute distal femur fractures and distal femur fracture nonunions.

Methods: This is a retrospective comparative study at a Level 1 academic trauma center, including 104 patients treated for acute distal femur fractures and 23 patients treated for distal femur nonunions between 2015 and 2019. The study compared dual plate fixation to other methods of fixation.

Results: In the acute fracture setting, the dual-plate construct had a shorter time to union (22.1 weeks vs. 29.5 weeks, p=0.1337) and a better union rate (100% vs. 73.6%, p=0.1848), though neither were statistically significant. Complication rates between dual plating and single lateral plating were similar (20% dual plate vs. 14.3% single lateral plate, p=0.7245). For femoral nonunions, both treatment groups achieved a 100% union rate. The time to union was slightly longer in patients treated with an adjunctive medial plate (35 weeks vs. 31 weeks, p=0.6207). However, medial adjunctive plating had a lower complication rate (0% vs. 26.7%, p=0.1081).

Conclusion: Dual plating plays a valuable role in the management of acute distal femur fractures, as well as in the adjunctive medial plating of distal femur nonunions treated with a lateral plate construct. This approach is especially beneficial for cases with inadequate medial cortical support or a high risk of varus collapse.

## Introduction

Distal femur fractures occur in a bimodal distribution of high-energy trauma in younger patients, and low-energy falls in the elderly population. Additionally, with the increasing use of total knee arthroplasty, incidence continues to surge. In the elderly population, bone quality, degree of comminution, and remaining distal bone stock have resulted in an increase in the use of locking plate constructs [1]. Studies have demonstrated mixed results on the efficacy of locked plate constructs, finding nonunion rates as high as 20% [2]. Nonunions of these fractures can be problematic and are associated with pain, decreased function, and substantial disability [3]. One theory for the incidence of nonunion associated with locking plates is that the constructs are too stiff and do not create a favorable environment for healing, in addition to malreduction or loss of reduction in the postoperative period [2,4]. However, due to the compromised bone quality and limited distal bone stock often associated with these fractures, locking plate technology is frequently required. In most cases, fixation is performed using a lateral approach alone [5].

Due to the issues associated with lateral locked plating, different techniques have been developed to provide a stable construct and improve the distribution of forces. These techniques include combining intramedullary nailing with lateral locked plating as a way to provide strength and maintain alignment [6]. Dual plate fixation of distal femur fractures has also been utilized, most commonly with complex fracture patterns or lack of a medial buttress [7-12]. Biomechanical evaluations of dual plating also show that the addition of a medial plate provides a more even load distribution, greater load to failure, and a smaller ultimate displacement of the construct [13,14]. To the best of our knowledge, only two studies directly compare distal femur fracture fixation utilizing dual plating versus single lateral plating [9,11], while other two studies demonstrate the addition of a medial plate following lateral locking plate fixation failure and nonunion, with mixed results [15,16]. We hypothesize that there is a role for medial plate fixation either as primary fixation in comminuted distal femur fractures or as salvage in nonunion/delayed union with previous lateral plating in isolation to give extra stability in association with bone graft.

## Materials and methods

A retrospective review was conducted at an urban Level 1 trauma center from January 2015 to August 2019 of patients treated for a distal femur fracture or distal femoral nonunion by one of five fellowship-trained orthopedic trauma surgeons. An IRB protocol was submitted to perform this retrospective analysis, which was approved for study under minimal risk (IRB# 1539398-1). Among the 391 patients identified, 111 met the inclusion criteria and received treatment for 104 distal femur fractures and 23 distal femoral nonunions. Sixteen of the distal femur nonunion patients were originally among the 104 acutely treated cases, while the remaining seven presented from an outside facility or had undergone initial treatment before the study period. A femur fracture was classified as non-union if it failed to heal radiographically within six to nine months of the initial procedure, showing either a lack of healing progress or construct failure, indicating the fracture would likely result in non-union.

Patients were sorted based on initial treatment instrumentation. Exclusion criteria for the study included patients under 18 years, patients with less than one-year follow-up that did not achieve union, operative fixation with an intramedullary device alone, and immediate operative treatment of fracture with distal femoral replacement.The minimum follow-up was four months if the union was achieved.

Patients were subdivided into three fixation categories: Single lateral locking plate, dual plate, and a nail-plate combination. Treatment of distal femoral nonunions was subdivided into two groups: Addition of a medial plate to an already existing lateral plate construct with or without bone grafting versus fixation through other methods, including exchange to a blade plate, addition of a nail to a plate construct, lateral exchange plating, and isolated bone grafting. The decision to dual plate a fracture or add an adjunctive medial plate in the presence of a nonunion was based upon surgeon preference, but it was generally reserved for fractures the operating surgeon thought required additional medial support.

All fractures were interpreted independently by two surgeons who were not involved with the patients, and a weighted kappa value was calculated to ensure interobserver agreement. Fractures were classified by the AO/OTA classification [17]. Open fractures were classified by the Gustilo-Anderson Classification [18]. Radiographic union was defined by bridging callus on three of four cortices. A fracture nonunion was defined as a lack of clinical or radiographic evidence of progression to union, with the opinion of the treating surgeon to have no chance of healing without additional intervention.

Treatment groups were compared to identify variables that could affect healing potential. Primary measured outcomes were union rate, time to union, and overall complication rate. Complications included superficial infections requiring oral antibiotics, deep infections requiring formal irrigation and debridement, and knee manipulation under anesthesia for a clinically limited range of motion. Rates of nonunion, hardware removal, amputation, and transition to total knee arthroplasty or distal femoral replacement were all considered separately. Secondary measured outcomes were blood loss, operating time, and average time to weight bearing as tolerated. Statistical comparisons were conducted utilizing a t-test for continuous variables and a Chi-squared test for categorical data.

## Results

Of the 111 patients that met the inclusion criteria, 41 were males, and 70 were females, with a mean age of 59.8 years. Sixteen patients treated for distal femur fractures at our center underwent revision surgery for nonunion, whereas the other seven nonunion patients were initially treated before 2015 or treated by an outside facility. Of the 104 distal femur fractures, 91 were treated with single lateral locking plates, five with dual plates, and eight with nail-plate combinations. The average follow-up time was 58 weeks, and no statistically significant difference was found when comparing initial patient demographics or fracture characteristics (Table 1)

**Table 1:** Demographic and fracture characteristics of distal femur fractures.

Variables	Lateral plate (LP)	Dual plate (DP)	Nail plate (NP)	p-value lateral plate vs. dual plate	p-value lateral plate vs. nail plate	p-value dual plate vs. nail plate
Patients	91	5	8			
Mean age, years (range)	61.5 (18-96)	52.8 (26-81)	60.8 (27-88)	0.5643	0.7151	0.7358
Sex				0.8658	0.4413	0.7249
Male, (%)	33 (36.3)	2 (40)	4 (50)			
Female (%)	58 (63.7)	3 (60)	4 (50)			
Nicotine use				0.4323	0.4858	0.8351
Yes (%)	34 (37.4)	1 (20)	2 (25)			
No (%)	57 (62.6)	4 (80)	6 (75)			
Pre-Injury ambulation				0.5532	0.5321	0.4106
Yes (%)	85 (93.4)	5 (100)	7 (87.5)			
No (%)	6 (6.6)	0 (0)	1 (12.5)			
Mean injury Severity Score (range)	11.7 (9-27)	15.75 (9-34)	15.6 (9-34)	0.5573	0.4723	0.9852
Mechanism				0.1589	0.9137	0.3878
Fall from ground (%)	54 (62.07)	2 (40)	5 (62.5)			
Motor vehicle accident (%)	28 (32.18)	2 (40)	3 (37.5)			
Gunshot wound (%)	2 (2.3)	1 (20)	0 (0)			
Fall from height (%)	3 (3.45)	0 (0)	0 (0)			
Isolated injury				0.7856	0.3661	0.7249
Yes (%)	60 (65.9)	3 (60)	4 (50)			
No (%)	31 (34.1)	2 (40)	4 (50)			
Ipsilateral injury				0.2112	0.1702	0.9282
Yes (%)	16 (17.6)	2 (40)	3 (37.5)			
No (%)	75 (82.4)	3 (60)	5 (62.5)			
Open vs. closed				0.1192	0.3562	0.0710
Open (%)	25 (27.5)	2 (40)	1 (12.5)			
Closed (%)	66 (72.5)	3 (60)	7 (87.5)			
Intra/extraarticular fracture				0.8622	0.7415	0.7249
Intraarticular (%)	51 (56)	3 (60)	4 (50)			
Extraarticular (%)	40 (44)	2 (40)	4 (50)			
Fracture classification				0.3684	0.0035	0.2196
33A1 (%)	4 (4.4)	1 (20)	2 (25)			
33A2 (%)	0 (0)	0 (0)	0 (0)			
33A3 (%)	9 (9.89)	0 (0)	2 (25)			
33B1 (%)	2 (2.2)	0 (0)	0 (0)			
33B2 (%)	0 (0)	0 (0)	1 (12.5)			
33B3 (%)	0 (0)	0 (0)	0 (0)			
33C1 (%)	1 (1.1)	0 (0)	0 (0)			
33C2 (%)	25 (27.47)	0 (0)	2 (25)			
33C3 (%)	23 (25.27)	3 (60)	1 (12.5)			

 Of the 23 distal femoral nonunions treated, eight were treated with the addition of medial plates with or without bone grafting, and 15 were treated via other methods (lateral blade plates, nail plates, intramedullary nails, and exchange lateral locking plates with or without bone grafting). The average follow-up was 69.9 weeks for nonunion patients, and we did not discover any statistically significant difference between the two groups regarding demographic or injury variables (Table 2).

**Table 2:** Demographic and fracture characteristics of distal femoral nonunions.

Variables	Medial plate	Other constructs	p-value
Patients	8	15	
Age, years	55.5 (20-85)	41.07 (25-72)	0.1077
Sex			0.4353
Male (%)	4 (50)	5 (33.3)	
Female (%)	4 (50)	10 (66.67)	
Nicotine use			0.8788
Yes (%)	4 (50)	7 (46.67)	
No (%)	4 (50)	8 (53.33)	
Pre-injury ambulation			1
Yes (%)	8 (100)	15 (100)	
No (%)	0 (0)	0 (0)	
Mechanism			0.9107
Fall from ground (%)	2 (25)	4 (28.57)	
Motor vehicle accident (%)	5 (62.5)	9 (64.29)	
Gunshot wound (%)	0 (0)	0 (0)	
Fall from height (%)	1 (12.5)	1 (7.14)	
Isolated injury			0.4023
Yes (%)	2 (25)	6 (42.86)	
No (%)	6 (75)	8 (57.14)	
Ipsilateral injury			0.5121
Yes (%)	4 (50)	5 (35.71)	
No (%)	4 (50)	9 (64.29)	
Open vs. closed			0.0195
Open (%)	3 (37.5)	12 (85.71)	
Closed (%)	5 (62.5)	2 (14.29)	
Intra/extraarticular fracture			0.0164
Intraarticular (%)	4 (50)	14 (93.33)	
Extraarticular (%)	4 (50)	1 (6.67)	
Fracture classification			0.1031
33A1 (%)	0 (0)	0 (0)	
33A2 (%)	0 (0)	0 (0)	
33A3 (%)	1 (12.5)	0 (0)	
33B1 (%)	0 (0)	0 (0)	
33B2 (%)	0 (0)	0 (0)	
33B3 (%)	0 (0)	0 (0)	
33C1 (%)	0 (0)	0 (0)	
33C2 (%)	2 (25)	9 (60)	
33C3 (%)	2 (25)	5 (33.3)	

Of note, pre-operative imaging and patient demographics were not available for all patients within the nonunion group.

Acute distal femur fractures

Primary outcome measures for single lateral locked plating, dual plating, and nail plating were not statistically significant, including rates of union, time to union, and complication rates (Table 3).

**Table 3:** Outcomes for distal femur fractures.

Variables	Lateral plate (LP)	Dual plate (DP)	Nail plate (NP)	p-value lateral plate vs. dual plate	p-value lateral plate vs. nail plate	p-value dual plate vs. nail plate
Operating room time (min)	162.5	194.7	282.8	0.0294^*^	0.0013	0.0040
Blood loss (mL)	186	150	337.5	0.3560	0.1899	0.1258
Time to weight bearing as tolerated (weeks)	13.5	8.4	9.43	0.1098	0.2377	0.7980
Radiographic union				0.1848	0.0951	1
Yes (%)	67 (73.6)	5 (100)	8 (100)			
No (%)	24 (26.4)	0 (0)	0 (0)			
Time to union (weeks)	29.5	22.1	39.9	0.1337	0.3041	0.117
Complication rate				0.7245	0.0872	0.5060
Yes (%)	13 (14.3)	1 (20)	3 (37.5)			
No (%)	78 (85.7)	4 (80)	5 (62.5)			

**Figure 1:**
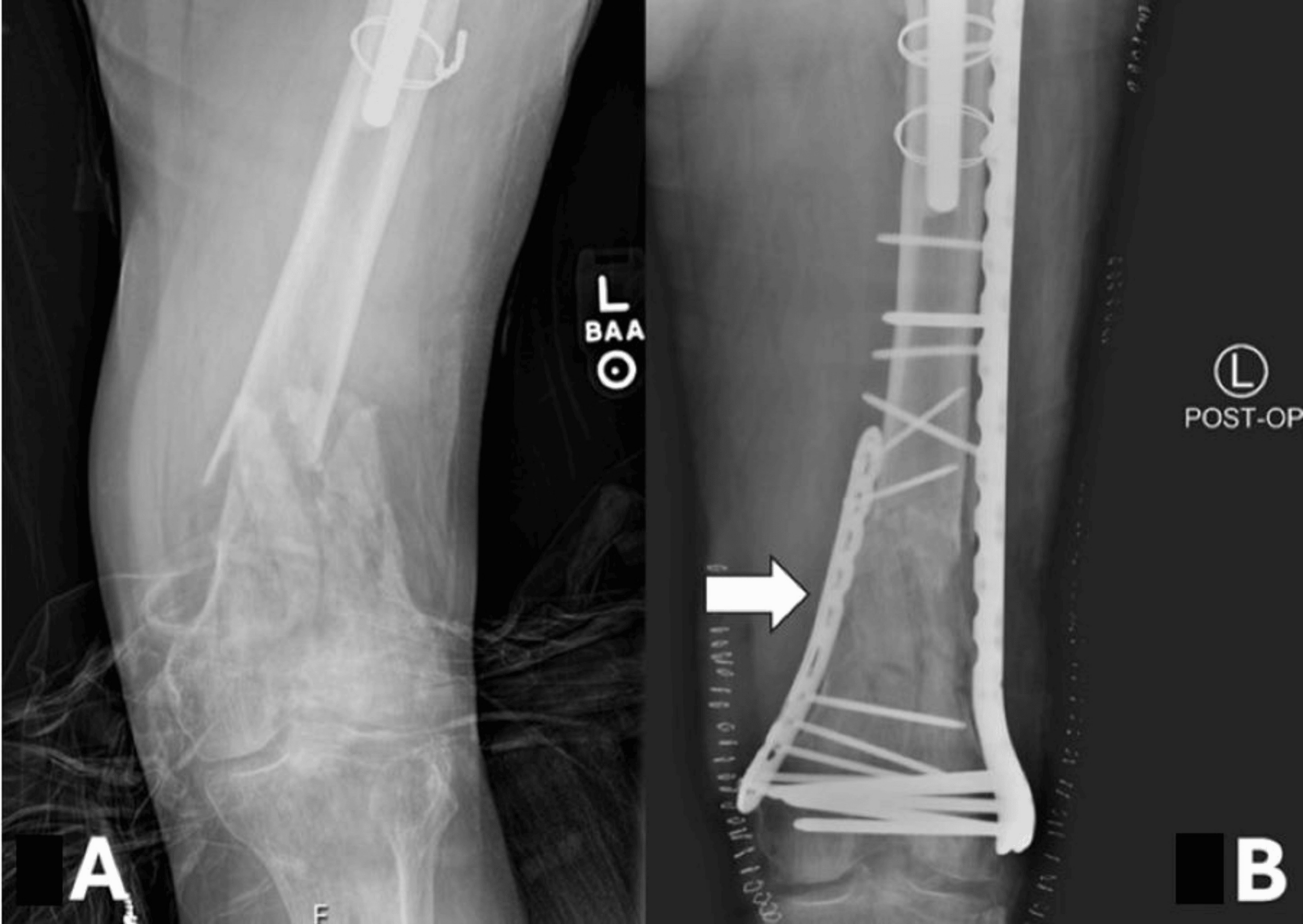
Distal femur and fracture fixation. A: Distal femur fracture; B: definitive fixation with lateral and medial plate. White arrow: medial plate providing additional fixation to the medial column in a dual plate construct.

All patients treated with dual plating and nail plating achieved union at an average of 22.1 weeks and 39.9 weeks, respectively. Single lateral locking plate resulted in a 26.4% nonunion rate with an average time to union of 29.5 weeks. However, the difference in union rates was statistically significant. Of the 24 patients that went on to nonunion, 18 underwent revision surgery. Nail plating had the highest complication rate, with 37.5% requiring oral antibiotics for superficial infections, though not statistically significant. Notably, for the dual plating construct group, 20% required manipulation under anesthesia for clinical knee stiffness. Finally, 14.3% in the single lateral locking plate construct group sustained a complication. Of these, four required oral antibiotics, seven required formal irrigation and debridement, and two underwent manipulation under anesthesia. The average operating time between the three groups was significantly different, with single lateral plating averaging 32.2 minutes shorter than dual plating. There were no significant differences in the other secondary outcomes, including average blood loss and average time to weight-bearing as tolerated. An example of dual plating can be seen in Figure 1.

Distal femoral nonunions

Of the 23 patients treated for distal femoral nonunions, 21 were initially treated with a single lateral locking plate, 1 with a nail plate, and 1 with a dual plate that was removed after a presumed union and subsequently collapsed into valgus. There were no statistically significant differences in primary or secondary outcomes between the two treatment groups (Table 4).

**Table 4:** Outcomes for distal femoral nonunions.

Variables	Medial plate	Other constructs	p-value
Operating room time (min)	180.5	222.7	0.1376
Blood loss (mL)	240.6	353.6	0.2657
Time to weight bearing as tolerated (weeks)	6.89	9.2	0.5702
Radiographic union			1
Union (%)	8 (100)	15	
Nonunion (%)	0 (0)	0	
Time to union (weeks)	35	31	0.6207
Complication rate			0.1081
Yes (%)	0 (0)	4 (26.67)	
No (%)	8 (100)	11 (73.33)	

**Figure 2:**
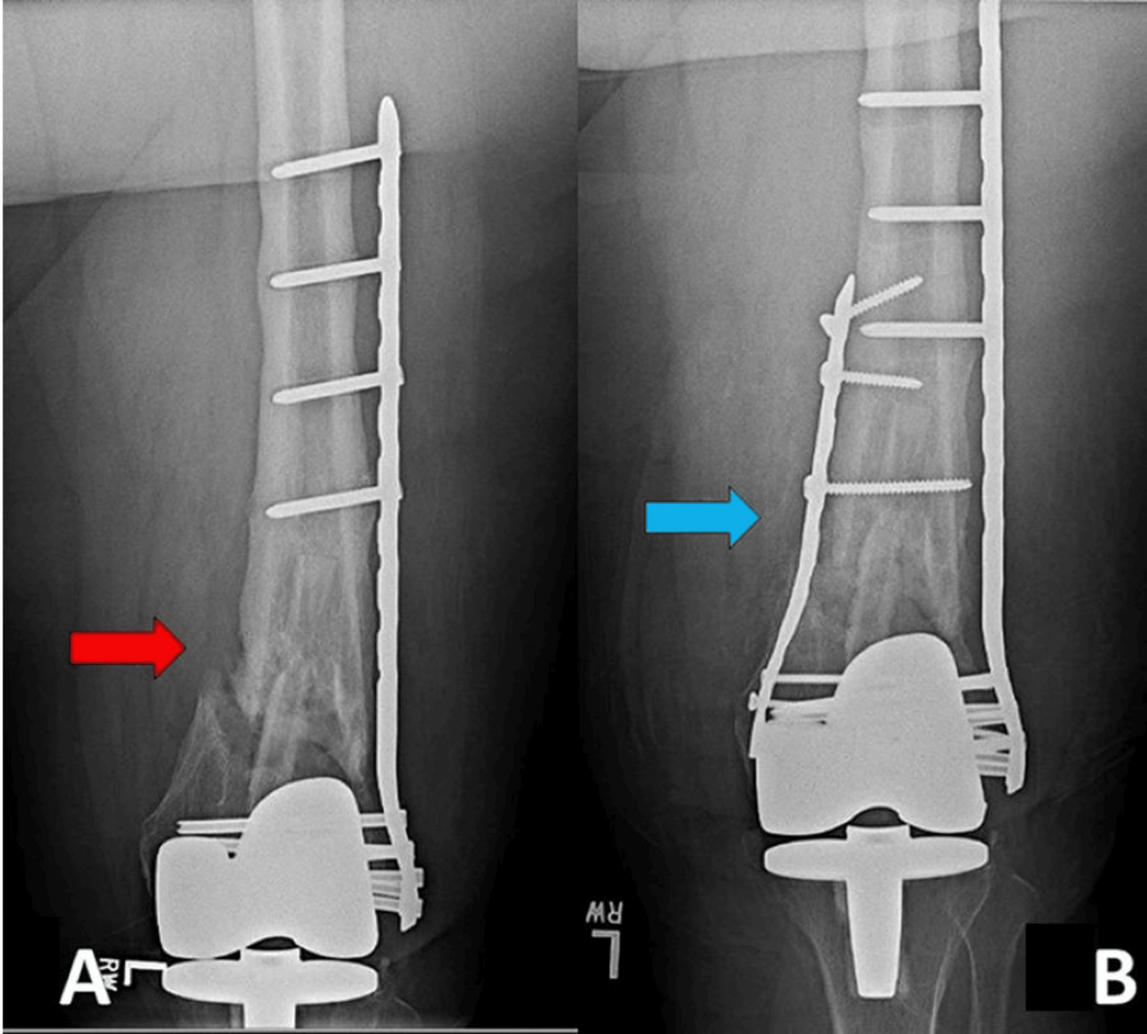
Distal femoral nonunion fracture. A: distal femoral periprosthetic nonunion with a single lateral locking plate; B: treatment with the addition of a medial plate. Red arrow: nonunion site within the metaphyseal region of a periprosthetic distal femur fracture previously treated with a lateral locking plate; Blue arrow: medial plate and bone graft added for additional fixation in a revision open reduction internal fixation of a periprosthetic distal femur nonunion.

All 23 patients achieved radiographic union. The patients treated with the addition of a medial plate averaged 35 weeks to the union, and other treatment modalities averaged 31 weeks to the union. Patients treated with adjunctive medial plating did not have any complications, but one patient required a staged Masquelet technique for a nonunion with significant bone loss. Of the patients treated with constructs other than medial adjunctive plating, four of 15 (26.7%) experienced complications, with three patients requiring irrigation and debridement and one patient requiring manipulation. Additionally, three patients required multiple revision surgeries for bone grafting or implantation of new hardware for continued nonunion in this group. An example of adjunctive medial plating for distal femur nonunion can be seen in Figure 2.

## Discussion

Fixed angle constructs provide stability in osteoporotic bone, especially in multifragmentary and intraarticular fractures that are not amenable to fixation via intramedullary nailing [4]. Despite the advantages of fixed-angle plates, nonunion rates of 20% have been reported [2]. Risk factors such as tobacco use, diabetes, increased body mass index, infection, and open fractures have been associated with nonunion [19,20]. One additional theory is that locked plating is too stiff and creates an uneven distribution of forces across the fracture that decreases bony healing and promotes an asymmetric callus [21-23]. Unstable fracture patterns that are highly comminuted with poor bone quality and loss of bone stock have benefited from fixation through dual plating and a nail-plate combination [7-12,24]. Biomechanical studies have supported the notion that these constructs provide superior fixation, strength, and more even load distribution as compared to a single locked lateral plate [13,14,25]. 

Dual plating in the treatment of distal femur fractures has been suggested as a treatment option for comminuted fractures with poor bone quality that are potentially prone to varus collapse. Though it does increase the stiffness of the construct, applying a medial plate as an augment to lateral plate fixation has demonstrated satisfactory outcomes for the treatment of distal femur fractures [8-11,13,26]. Sanders et al. applied a medial plate and autologous bone graft to a lateral condylar buttress plate to intraarticular fractures that were intraoperatively noted to experience varus collapse of the distal fragment [26]. Bai et al. utilized similar criteria to Sanders and conducted a varus stress test intraoperatively, adding a medial plate when medial support was needed, resulting in similar union rates and time to union [11]. Steinberg et al. applied a dual plate construct to patients with distal femur nonunions and distal femur fractures with poor bone quality, significant comminution, and fractures close to the joint line [7]. Thirty-two patients were treated with an average time to radiographic union of 12 weeks, with the exception of one patient who required bone grafting and another patient who refracted [13]. Recently, Bologna et al. completed a retrospective review of 21 comminuted distal femur fractures and compared outcomes between single lateral locked plating and dual plating. All eight patients that were dual-plated achieved union significantly faster than single-plated fractures [9]. The results of our study support the efficacy of a dual plate construct with a 100% union rate and an average time to union of 22.1 weeks. Although not statistically significant due to limited power, this is comparable to the 73.6% union rate observed in the single lateral plate group, which had an average time to union of 29.5 weeks.

The nail-plate combination has recently been applied to treat distal femur fractures in patients with unstable fractures that would benefit from early mobilization and weight bearing. Liporace et al. demonstrated that nail-plate constructs could allow patients to weight-bear immediately after surgery and postulate better functional outcomes compared to prolonged immobilization [6]. Additionally, nail-plate combinations have been successfully applied to periprosthetic fractures, even in the setting of ipsilateral THA and TKA [24]. In our study, all eight patients treated with a nail and plate achieved radiographic union, similar to the dual plate construct group. The superficial infection complication rate in this group was 37.5% (3/8), which could be from the additional hardware and procedural time or a skew in the data due to a limited number of patients. 

There is debate as to what surgical technique is most successful in achieving union for a distal femur nonunion. Medial plate application has been shown to be a viable treatment option for distal femoral nonunions that does not compromise a large portion of distal bone stock and provides stable medial support. Chapman and Finkemeier retrospectively reviewed 18 patients with distal femoral nonunions and reported on the efficacy of a suggested treatment algorithm [27]. Thirteen patients of the 18 were treated with a dual plate construct consisting of a medial plate with either a large fragment plate, a fixed angle device, or a condylar buttress plate. All 18 patients achieved union by the time of the last follow-up, with a reported complication rate of 17%. Ebraheim et al. studied 14 patients who experienced a distal femur nonunion after a single lateral locking plate fixation. Nonunions were treated by leaving the lateral locking compression plate in situ and augmenting with additional screws, a medial plate, and/or some form of bone grafting or biologic substitute. However, only eight of 14 (57%) patients achieved union in the study [16]. Holzman et al. treated 23 distal femoral nonunions with the addition of a medial plate and autogenous bone grafting through a single or two-stage procedure [15]. Ninety-five percent of patients achieved union by 12 months.

Another approach in the management of distal femoral nonunions is the use of a nail and plate in combination. Studies have evaluated the effectiveness of this treatment in scenarios where a new plate and nail are implanted, or a lateral plate is added to augment an already implanted nail [28,29]. Attum et al. retrospectively reviewed 10 patients who were treated with a nail plate construct after failed initial fixation of a lateral plate or retrograde intramedullary nail [28]. They reported a 100% union rate and had minimal complications, with one patient (10%) with a deep infection that required reoperation. A study assessing the addition of a lateral plate or fixed-angle constructs to an in situ intramedullary nail reported a 100% union rate in treated femoral nonunions [29].

In our study, the addition of a medial plate to a lateral locking plate for the treatment of a distal femoral nonunion resulted in all eight patients achieving radiographic union in an average of 35 weeks with no significant complications. All patients who were treated by fixation devices other than a dual plate construct achieved radiographic union in an average of 31 weeks. Within this group, four of 15 patients (26.7%) experienced complications, three of which were continued nonunion. 

Limitations

The limitations of this study include the retrospective nature of the study and the limited number of patients in the study treated with dual plating and nail-plating. The study does demonstrate differences between single lateral plating, dual plating, and nail-plating in the acute fracture setting. However, being underpowered, many of those differences were not statistically significant. Additionally, the limited follow-up of some patients despite ongoing complications and nonunions could have changed the data if those patients had undergone revision surgeries and eventually achieved union.

## Conclusions

Given the findings in this retrospective study, the authors believe there is a role for dual plating of acute distal femur fractures, as well as adjunctive medial plate application to distal femur nonunion treated with a lateral plate construct. These techniques show promising results in terms of achieving union with minimal complications. Larger prospective studies could shed more light on the role of these techniques.
